# Autosomal dominant sleep-related hypermotor epilepsy associated with a novel mutation of *KCNT1*

**DOI:** 10.1515/tnsci-2022-0241

**Published:** 2022-08-30

**Authors:** Jinyu Lu, Gaohua Zhao, Dayao Lv, Lanxiao Cao, Guohua Zhao

**Affiliations:** Department of Neurology, The Fourth Affiliated Hospital, Zhejiang University School of Medicine, Yiwu, China

**Keywords:** autosomal dominant sleep-related hypermotor epilepsy, clinical features, *KCNT1*, mutation

## Abstract

Autosomal dominant sleep-related hypermotor epilepsy (ADSHE) is characterized by severe sleep-related rigid hypermotor seizures. The pathogenic genes of ADSHE include genes encoding subunits of the neuronal nicotinic acetylcholine receptor, *KCNT1*, *DEPDC5*, *NPRL2/3*, *CABP4*, and *CRH.* Individuals with *KCNT1*-related ADSHE are more likely to develop seizures at a younger age, have cognitive comorbidity, and display psychiatric and behavioral problems. In this study, a 12-year-old Chinese girl was referred for genetic evaluation of grand mal seizures. She had paroxysmal convulsions of the limbs and loss of consciousness just after falling asleep without obvious triggers. A novel heterozygous missense mutation c.2797C > T (p.Arg933Cys) in exon 24 of the *KCNT1* was identified in the proband by whole-exome sequencing and Sanger sequencing, and the clinical symptoms were compatible with ADSHE. The proband’s father has been showing similar symptoms for more than 20 years and had the same site mutation. Her mother and sister were physically and genetically normal. The study revealed a novel variant in the *KCNT1* and expanded the mutation spectrum for this clinical condition. Our results provide further evidence supporting a causative role in *KCNT1* variants in ADSHE.

## 1 Introduction

Sleep-related hypermotor epilepsy (SHE), formerly known as nocturnal frontal lobe epilepsy, is characterized by focal seizures with various motor manifestations during sleep. It is an idiopathic focal epilepsy syndrome that occurs during nonrapid eye movement (REM) sleep. SHE has a range of clinical manifestations, from brief, stereotyped, and sudden arousal to more complex dystonia, which can be specifically manifested as paroxysmal arousal, nocturnal paroxysmal dystonia, and intermittent loitering [1]. SHE includes both sporadic and familial forms. Autosomal dominant sleep-related hypermotor epilepsy (ADSHE) belongs to familial SHE. ADSHE was first described in 1981 by Lugaresi and Cirignotta, who first named it “hypnotic paroxysmal dystonia” [2]. Scheffer et al. further defined the syndrome as a form of frontal lobe epilepsy and described its familial nature, with clusters of motor symptoms at night as its prominent clinical feature [3]. To improve the definition of this disorder and establish diagnostic criteria with levels of certainty, it was named SHE in Bologna in September 2014 [4]. As a genetic variation of SHE, ADSHE itself is clinically and biologically heterogeneous [1]. In 1995, Steinlein et al. discovered the first pathogenic gene for ADSHE, namely, *CHRNA4*, which encodes one of the subunits of the neuronal nicotinic acetylcholine receptors [5,6]. Fifteen years later, *KCNT1* was identified as the major disease-causing gene of epilepsy of infancy with migrating focal seizures (EIMFS) [7]. In addition, other genes were implicated in the disease, including *DEPDC5*, *NPRL2/3*, *CABP4*, and *CRH* [8].

Worldwide, more than 60 mutations of the *KCNT1* have been found to be associated with various types of epilepsy. Herein, we describe a case of ADSHE with the typical clinical manifestations and a *KCNT1* mutation (c.2797 C > T, p.Arg933Cys), and this specific mutation was not previously reported in patients.

## 2 Case reports

Proband, female, 12 years old (II:1, Figure 1a), visited our outpatient clinic in December 2020 for 1-year history of repeated convulsions and loss of consciousness. In November 2019, the patient began to have paroxysmal convulsions of the limbs and loss of consciousness just after falling asleep without obvious triggers. The convulsions lasted for about 1 min and were not associated with fecal incontinence or foaming at the mouth, and she recovered consciousness in about 10 min. No special treatment was provided. Since then, she has been experiencing occasional daytime lower limb twitching for several seconds without loss of consciousness. She was not on any medication. In June 2020, the patient presented with convulsions and loss of consciousness just after falling asleep again and was diagnosed with epilepsy. She was prescribed 0.5 g levetiracetam twice a day. The patient’s symptoms did not improve and repeated leg twitches occurred during the day. Levetiracetam was associated with irritability, so its dose was gradually decreased and oxcarbazepine was added. The patient is now taking 0.3 g oxcarbazepine twice a day. Her seizures have not recurred for nearly 2 years, and she has not had any oxcarbazepine-related side effects.

**Figure 1:**
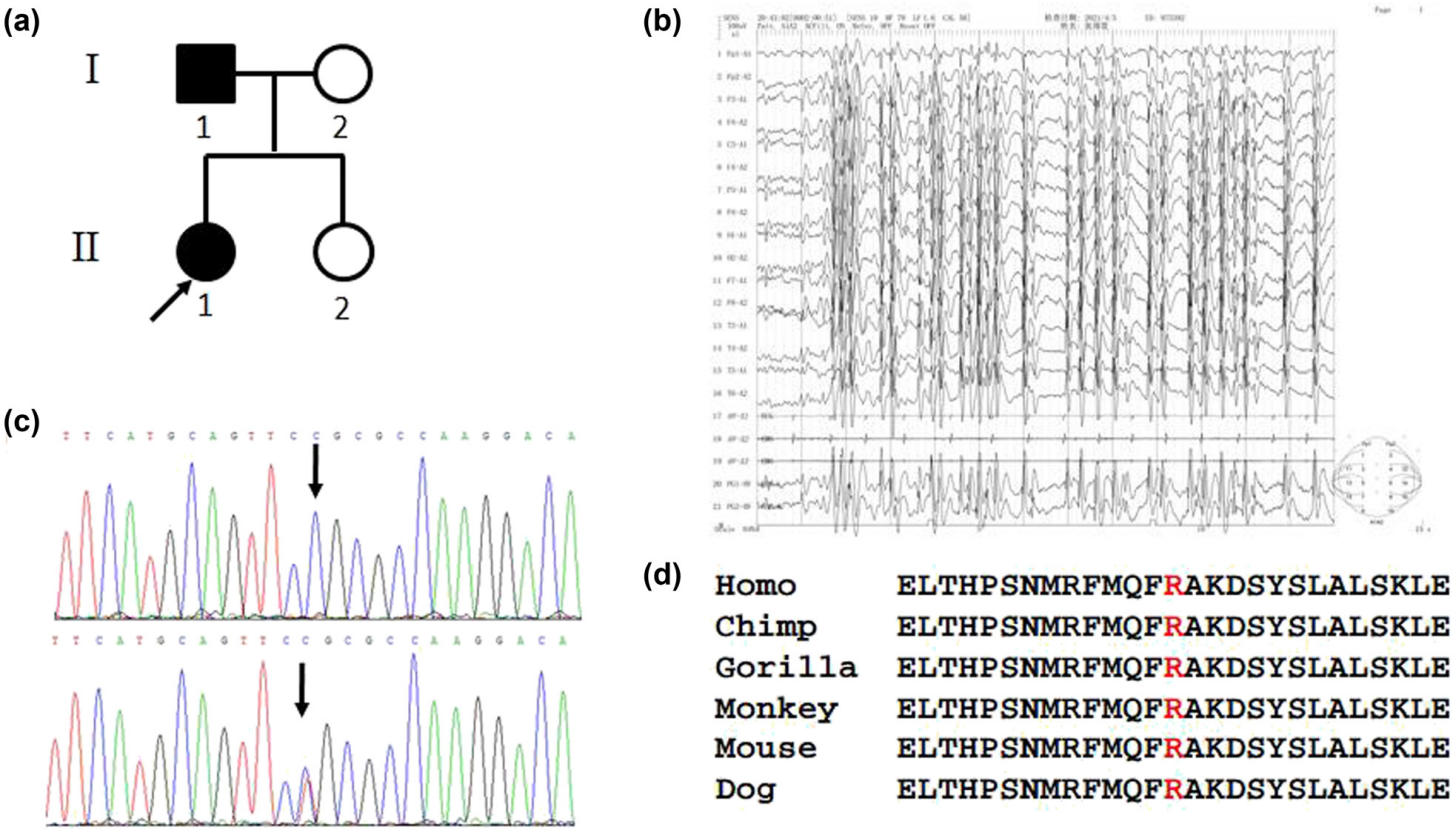
(a) Pedigree of the proband. (b) The results of video EEG. Many 2- to 3-Hz sharp-slow wave and spike-slow wave complex paroxysms were detected on both sides when the patient was awake and asleep. (c) The control sequence (upper) and the mutant sequence (lower). (d) Conservation analysis of the amino acid in different species.

The proband’s past, personal, and reproductive history was unremarkable. Neurological examination showed no positive signs. The results of routine and biochemical tests, as well as cranial magnetic resonance imaging (MRI), were normal. Video electroencephalograph (EEG) showed many 2- to 3-Hz sharp-slow wave and spike-slow wave complex paroxysms on both sides when she was awake and asleep; the paroxysms sometimes occurred laterally and obviously on the right side. Epileptic activity phase inversion was occasionally observed in the two frontotemporal regions of the bipolar leads. According to electrical status epilepticus of sleep, the discharge index was >85% (Figure 1b).

Proband’s father, 37 years old, had a more than 20-year history of recurrent convulsions of the limbs lasting for 1–2 min that mainly occurred at night and occasionally during the day and were accompanied by loss of consciousness. He was diagnosed with epilepsy, which was under control with valproate and carbamazepine. He had not taken oxcarbazepine. The proband’s mother (36 years old) and younger sister (10 years old) were healthy.

The proband and family members I:1, I:2, II:1, and II:2 (Figure 1a) were enrolled after giving written consent. Genomic DNA was extracted from white blood cells using a standard method (QIAGEN, Hilden, Germany). Whole exome sequencing (KRD Biomedical Group, Hangzhou, China) was performed in the proband. The identified variants were used for cosegregation analysis in the family members I:1, I:2, II:1, and II:2 by Sanger sequencing, and polymorphism was excluded by 500 healthy controls. Genetic analysis showed *KCNT1* mutation c.2797 C > T (p.Arg933Cys) (Figure 1c) in family members I:1 and II:1, which was absent in family members I:2 and II:2. This variant was not present in 500 healthy controls and predicted to be deleterious by SIFT software and probably damaging by Polyphen-2 software, suggesting that this variant was responsible for the patients’ phenotype. Conservation analysis showed that p.Arg933 was conserved in different species (Figure 1d). The variant (c.2797 C > T) was found to be present in population databases (rs150395210, ExAC 0.01%) but has not been reported in individuals with KCNT1-related disease. Therefore, this mutation is being reported for the first time in a patient with ADSHE.


**Ethical approval:** The research related to human use has been complied with all the relevant national regulations, institutional policies and in accordance the tenets of the Helsinki Declaration, and has been approved by the Ethical Committee of The Fourth Affiliated Hospital, Zhejiang University School of Medicine.
**Informed consent:** Informed consent was obtained from the family members and control subjects.

## 3 Discussion

Herein, we describe members of a Chinese family with a novel *KCNT1* mutation. According to the patient’s clinical manifestations, family history, and auxiliary examination results, the clinical diagnosis of the proband was considered ADSHE.

ADSHE is a type of focal epilepsy with onset ages ranging from 2 months to 52 years, usually occurring in early childhood and adolescence, averaging 11.7 years [9,10]. ADSHE is characterized by sleep-related rigid hypermotor seizures that usually occur during non-REM sleep, including in stage 2 and stage 3–4 [11]. SHE affects both sexes but predominates in males [4,12]. The sex-dependent incidence of ADSHE has been less mentioned, only in earlier studies mentioning that male and female are equally represented [11]. Fifty-eight percent of attacks occur shortly after falling asleep, 48% occur in the early morning, 9% throughout the night, and 30% during daytime naps. The average seizure duration was about 74 s, so seizures are generally short lived. Episodes begin with wheezing, moaning, or uttering single words, which follows with somatokinetic automatism, such as sitting up suddenly and moving vigorously. Most patients can retain consciousness during the attack but cannot control their behaviors. Some patients lost consciousness during the attack, indicating a secondarily generalized epileptic seizure. Patients also exhibit several auras such as systemic tingling or trembling, auditory hallucinations, and photosensitive rings in the head or limbs [11]. Stress, sleep deprivation, and menstruation are triggers for seizures in one-fifth of cases. At the time of seizure, seizures in almost all patients were accompanied by manifestations of autonomic dysfunction, including heart rate, respiration, vasomotor tone, and sympathetic skin responses. In particular, tachycardia occurs simultaneously with seizures in approximately 90% of patients [1,5].

Currently, diagnosis mainly depends on history and clinical symptoms. Seizure sightings accompanied by core symptoms can be confirmed with a suspected diagnosis of SHE. Focal brain computed tomography and/or MRI abnormalities were present in a few cases. Video EGG recording is an essential tool for diagnosis and differential diagnosis and is necessary to analyze and compare symptomatology of attacks to confirm individual attack patterns [1,4,12,13]. Most patients have normal daytime EEG, and more than half have normal interictal EEG during sleep. A few ictal EEG recordings showed clear epileptic activity (spikes and waves), but diffuse (background flattening) or focal EEG activity (rhythmic *θ* or *δ* activity prominent in the front quadrant) could be recorded in the majority (55%) of subjects [5,14]. Epileptic seizures in ADSHE patients showed the EEG pattern of frontal lobe epilepsy (paroxysmal bursts of slow and sharp waves dominated by the frontal lobe) [10]. This is consistent with our proband’s clinical features. The level of consciousness during and after seizures is not a key clinical symptom for diagnosis [12].

The severity of epileptic seizures varied widely within families. There are also considerable individual differences in severity at different stages of life, which is related to age. The duration and frequency of seizures are long and frequent in children and adolescents, and they tend to decrease in complexity and frequency in adults, although they rarely disappear completely [11]. The mild ones can be asymptomatic or mild without medical intervention or can be well controlled only with carbamazepine [15], while the severe ones can also have mental, behavioral, and cognitive impairment, intellectual disability, and developmental degradation in addition to motor symptoms [16]. This may be due to the fact that ADSHE is not a purely single-gene disease and other factors (genetic, epigenetic, and/or environmental) interact with major genes to produce a specific phenotype [17,18]. Epilepsy without underlying brain disease and completely sleep-related seizures have a better prognosis.

The clinical manifestations of the cases we collected were mainly limb convulsions and loss of consciousness during sleep at night, with occasional limb convulsions, which were consistent with the clinical manifestations of ADSHE. Co-segregation analysis indicated that the patient was heterozygous for the c.2797C > T (p.Arg933Cys) mutation in the *KCNT1*. Ethnic similarity analysis suggested that the corresponding amino acid was conserved. Another pathogenic *KCNT1* mutation in the same site as this proband (c.2797C > G, R933G) was previously reported in a Chinese child who had intractable seizures starting 3 days after birth and severe developmental delay and was finally diagnosed with EIMFS [19]. The same *KCNT1* mutations can be associated with different phenotypes, even in individuals within the same family [20]. Additional mechanisms (i.e., effect of modifier genes, environmental factors, or both) are likely to influence the pleiotropy, variable expressivity, and incomplete penetrance associated with mutations of *KCNT1* [20]. Our study expanded the gene clinical spectrum of *KCNT1*.

**Figure 2:**
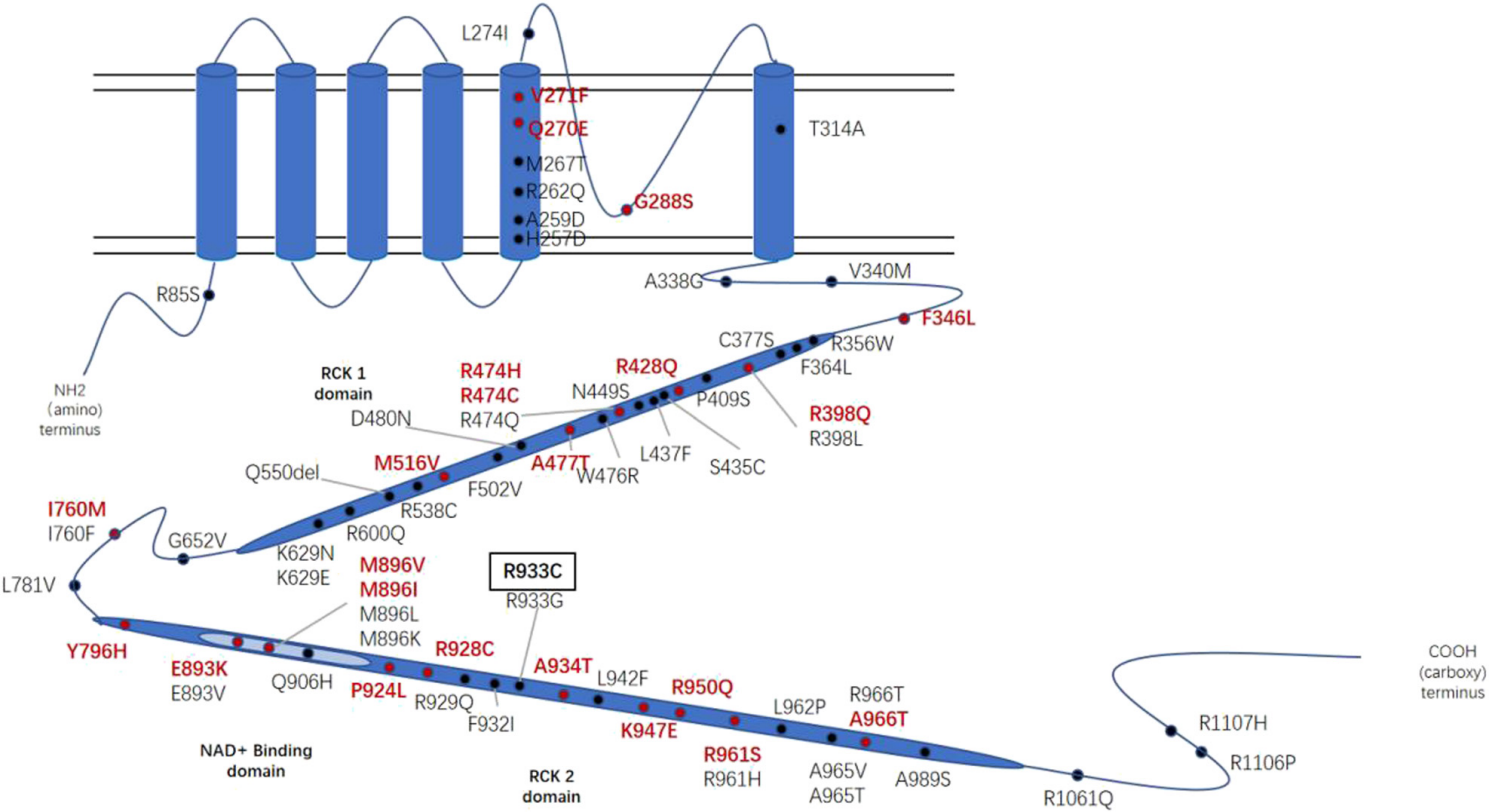
Structure of the slack protein, which is encoded by the *KCNT1*. It comprises six transmembrane domains (S1–S6) with a pore-forming region between S5 and S6. The intracellular domains in the C terminus are named RCK1 and RCK2, and the latter contains the NADP-binding domain. Pathogenic variants from both the literature and our case are shown: previously published (in black), our variant (with the square), and hotspot sites (in red and highlighted).

Human *KCNT1* is located on chromosome 9q34.3 and consists of 31 exons. The gene is transcribed to produce a 4.7 kb mRNA encoding a 138kDA protein that is a sodium-gated potassium channel and is the largest known potassium channel subunit [21]. It forms tetramers comprising six transmembrane domains with a pore-forming region, regulator of potassium conductance (RCK), and nicotinamide adenine dinucleotide binding domains. The expression profile of human mRNA showed high expression in most brain regions except corpus callosum and substantia nigra, playing an important role in regulating neuronal excitability [22,23]. We consider that changes in channel structure lead to abnormal neuron discharge, but further studies are needed to prove the functional changes. Mutations of *KCNT1* lead to severe early-onset epilepsy, usually accompanied by other comorbidities [24]. A total of 69 mutations have been identified (Figure 2). These mutations correspond to multiple phenotypes, including EIMFS, ADSHE, delayed myelination, leukodystrophy, leukoencephalopathy, Ohtahara syndrome, West syndrome, and other disorders. EIMFS and ADSHE are two of the most common phenotypes [25]. Individuals with *KCNT1*-related ADSHE are more likely to develop seizures at a younger age, have cognitive comorbidity, and display psychiatric and behavioral problems than individuals with ADSHE due to other causes [25]. The same familial *KCNT1* mutation was reported in two families with SHE and EIMFS, which suggests that additional factors modify the phenotype [18,26].

Epilepsy is relatively well tolerated for most SHE and ADSHE patients because they tend to be nocturnal. Carbamazepine is the preferred drug for nighttime seizures [1,5], which is usually effective at a low dosage [1] and can reduce seizures by about 20–50% in patients [27]. Symptoms usually recur when the drug is stopped. Carbamazepine metabolites, however, can produce serious toxic side effects. The less toxic second-generation compound, oxcarbazepine, has shown good efficacy in ADSHE patients, including some who are insensitive to other drugs [12]. In the case we collected, the treatment effect of levetiracetam was not good, but symptoms were controlled with the addition of oxcarbazepine.

Studies have shown that despite a partial reduction in nocturnal seizures, standard antiepileptic therapy is not particularly effective in reducing sleep instability compared to preconditioning [28]. About one-third of the patients had poor response to drug treatment [8]. Usually, patients with a high frequency of seizures are resistant. For patients with drug-resistant SHE, surgical intervention can be carried out, which has a good effect. More than two-thirds of SHE patients are completely controlled after surgery, and the frequency and intensity of seizures in the remaining patients are significantly reduced [12]. In the case of response to treatment, seizures often recur after withdrawal of anti-epileptic drugs [1]. Reducing factors that promote seizures, such as sleep instability, could lead to more effective treatment strategies.

In conclusion, a heterozygous missense mutation in exon 24 of the *KCNT1*, c.2797 C > T p.Arg933Cys, was detected by gene sequencing in a family with ADSHE. This is the first time this variant has been reported in association with clinically diagnosed ADSHE. The symptoms of ADSHE caused by the *KCNT1* mutation are serious, and the onset age is early. Therefore, gene detection in the early stage and rapid identification of gene variations would be of great significance for its diagnosis, treatment, and prognosis.
